# Identification of susceptibility loci for cardiovascular disease in adults with hypertension, diabetes, and dyslipidemia

**DOI:** 10.1186/s12967-021-02751-3

**Published:** 2021-02-25

**Authors:** Youhyun Song, Ja-Eun Choi, Yu-Jin Kwon, Hyuk-Jae Chang, Jung Oh Kim, Da-Hyun Park, Jae-Min Park, Seong-Jin Kim, Ji Won Lee, Kyung-Won Hong

**Affiliations:** 1 Department of Family Medicine, Gangnam Severance Hospital, Yonsei University College of Medicine, 211, Eonju‐ro, Gangnam‐gu, Seoul, 06273 Korea; 2 Healthcare R&D Division, Theragen Bio Co., Ltd., Gwanggyo-ro 145, Suwon-si, Gyeonggi-do 16229 Republic of Korea; 3 Department of Family Medicine, Yongin Severance Hospital, Yonsei University College of Medicine, 363, Dongbaekjukjeon-daero, Giheung-gu, Yongin-si, 16995 Gyeonggi-do Korea; 4 Division of Cardiology, Severance Cardiovascular Hospital, Yonsei University College of Medicine, 50-1, Yonsei-ro, Seodaemun-gu, Seoul, 03722 Korea

**Keywords:** Hypertension, Diabetes mellitus, Dyslipidemia, Cardiovascular disease, Genome-wide association studies

## Abstract

**Background:**

Hypertension (HTN), diabetes mellitus (DM), and dyslipidemia (DL) are well-known risk factors of cardiovascular disease (CVD), but not all patients develop CVDs. Studies have been limited investigating genetic risk of CVDs specific to individuals with metabolic diseases. This study aimed to identify disease-specific and/or common genetic loci associated with CVD susceptibility in chronic metabolic disease patients.

**Methods:**

We conducted a genome-wide association study (GWAS) of a multiple case–control design with data from the City Cohort within Health EXAminees subcohort of the Korean Genome and Epidemiology Study (KoGES_HEXA). KoGES_HEXA is a population-based prospective cohort of 173,357 urban Korean adults that had health examinations at medical centers. 42,393 participants (16,309 HTN; 5,314 DM; 20,770 DL) were analyzed, and each metabolic disease group was divided into three CVD case-controls: coronary artery disease (CAD), ischemic stroke (IS), and cardio-cerebrovascular disease (CCD). GWASs were conducted for each case–control group with 7,975,321 imputed single nucleotide polymorphisms using the Phase 3 Asian panel from 1000 Genomes Project, by logistic regression and controlled for confounding variables. Genome-wide significant levels were implemented to identify important susceptibility loci.

**Results:**

Totaling 42,393 individuals, this study included 16,309 HTN (mean age [SD], 57.28 [7.45]; 816 CAD, 398 IS, and 1,185 CCD cases), 5,314 DM (57.79 [7.39]; 361 CAD, 153 IS, and 497 CCD cases), and 20,770 DL patients (55.34 [7.63]; 768 CAD, 295 IS, and 1,039 CCD cases). Six genome-wide significant CVD risk loci were identified, with relatively large effect sizes: 1 locus in HTN (HTN-CAD: 17q25.3/*CBX8-CBX4* [OR, 2.607; *P* = 6.37 × 10^−9^]), 2 in DM (DM-IS: 4q32.3/*MARCH1-LINC01207* [OR, 5.587; *P* = 1.34 × 10^−8^], and DM-CCD: 17q25.3/*RPTOR* [OR, 3.511; *P* = 1.99 × 10^−8^]), and 3 in DL (DL-CAD: 9q22.2/*UNQ6494-LOC101927847* [OR, 2.282; *P* = 7.78 × 10^−9^], DL-IS: 3p22.1/*ULK4* [OR, 2.162; *P* = 2.97 × 10^−8^], and DL-CCD: 2p22.2/*CYP1B1-CYP1B1-AS1* [OR, 2.027; *P* = 4.24 × 10^−8^]).

**Conclusions:**

This study identified 6 susceptibility loci and positional candidate genes for CVDs in HTN, DM, and DL patients using an unprecedented study design. 1 locus (17q25.3) was commonly associated with CAD. These associations warrant validation in additional studies for potential therapeutic applications.

## Background

Cardiovascular disease (CVD) is the leading cause of death globally, and the prevalence of cardiovascular disease is constantly progressing in both developed and developing nations [1]. According to a World Health Organization report, an estimated 17.9 million people died from heart disease and stroke in 2016, representing 31% of all global deaths, and it was projected that about 23.6 million people will die from CVDs by 2030 [2]. In line with the global trend, economic and social developments have significantly impacted lifestyles of Koreans; CVDs are now one of the main causes of death in Korea, accounting for 1 in every 5 deaths [3]. Therefore, interventions to prevent and reduce cardiovascular risk factors are warranted.

Epidemiological evidence suggest that CVD is associated with age, sex, ethnicity, behavioral risk factors (smoking, alcohol use, and low physical activity levels), and several chronic metabolic diseases. Among them, hypertension (HTN), dyslipidemia (DL), and type 2 diabetes (DM) are the most common modifiable risk factors for CVD, which is known as the most prevalent cause of mortality and morbidity in populations with HTN, DM, and DL [4]. Approximately two-thirds of all adults with HTN at 30 years of age have a ~ 40% higher risk of experiencing a CVD event and systematic reviews have revealed that lowering blood pressure greatly reduced major CVD events and all-cause mortality, irrespective of the initial blood pressure [5]. Patients with DM had a 10% greater risk of coronary artery disease (CAD), a 53% greater risk of myocardial infarction (MI), and a 58% greater risk of stroke compared to those without diagnoses of DM, and CVD accounts for approximately half of all deaths in patients with DM, largely due to an increased risk of stroke and MI [6]. Further, in the INTERHEART study, 49% of the population-attributable risk of a first MI was contributed by DL, and the prevalence of DL was found to be between 83 and 87% in Asian patients with CVD [7].

There are various biochemical mechanisms that independently increase the risk of CVD in people with these chronic metabolic diseases. Lipid oxidation is thought to be an important determinant of atherosclerosis, which leads to CVD; moreover, high levels of low‐density lipoprotein (LDL) and low levels of high‐density lipoprotein (HDL) are associated with MI and stroke in DL [8]. Endothelial dysfunction, vascular inflammation, arterial remodeling, atherosclerosis, dyslipidemia, and obesity are common risk factors for CVD in HTN and DM. In addition, upregulation of the renin–angiotensin–aldosterone system, oxidative stress, inflammation, and activation of the immune system contribute to the close relationship between DM, HTN, and CVD [9].

However, only limited evidence is available regarding the relationship between multiple chronic metabolic diseases and CVDs. Furthermore, despite maintaining optimal levels of metabolic indices, such as blood pressure, glucose, or lipids, CVDs still remain prevalent in individuals with HTN, DM, and DL. Evidence suggest that although hyperglycemia contributes to ischemic events, it is not the only factor, because both pre-diabetes and normoglycemic patients show risk for most types of CVD [10]. High-dose statin therapy may provide some incremental benefit between 10 and 20%; nevertheless, statin-treated patients remain at high residual risk for future cardiovascular events [11]. This suggests that other non-modifiable risk factors, such as genetic and genomic variables, need to be considered as well.

Genetic factors are important contributors to the risk of CVDs; recent genome-wide association studies (GWASs) strongly support that genetic susceptibility to CVD is largely derived from gene mutations or variation [12]. Loci nearest a lead single nucleotide polymorphism (SNP) was shown to have genome-wide significant associations with obesity, blood pressure, lipids, DM, CAD, and stroke [13]. However, over the past decade, the majority of GWASs for CVDs or related risk factors have been conducted in populations with European white ancestry and limited evidence is available in Asian populations [14]. Furthermore, few analyses focused on the genetic differences in CVD susceptibility between individuals with the same chronic metabolic conditions.

Therefore, we conducted three sets of GWASs between case-controls of major CVDs in HTN, DM, and DL patient groups using data from the well-known nationwide Korean Genome and Epidemiology Study (KoGES). We aimed to find genetic pathways linking the development of CVD with each metabolic disease group; we further aimed to identify the potential presence of a common pathway leading to CVD among the commonly co-existing metabolic conditions.

## Methods

### Study populations

Our study used a part of the KoGES dataset obtained from the Korean Center for Disease Control and Prevention. The largest cohort of KoGES is the health examination cohort [KoGES_HEXA], and its dataset consists of participants’ medico-pharmacologic history, anthropometric traits, and blood biochemistry traits [15]. Briefly, KoGES_HEXA is a population-based prospective cohort of 173,357 urban Korean adults that had health examinations at medical centers, recruited from the national health examinee registry. Participants were men and women, aged 40–69 years from 14 major cities across Korea and recruited at baseline between 2004 and 2013. A total of 58,701 participants, whose genome-wide SNP genotype data were obtained, were included in the city-based cohort of the KoGES. All participants voluntarily signed an informed consent form before the study, and the study protocol was approved by the Institutional Review Boards (IRB) of the institutions that participated in KoGES. This study was performed in accordance with the Declaration of Helsinki and approved by the IRB of Theragen Etex (Approval Numbers: 700062-20190819-GP-006-02).

### Measurement of anthropometric and laboratory data & Definition of lifestyle factors

Study participants completed a standardized medical history and lifestyle questionnaire and underwent a comprehensive health examination by trained medical staff according to a standard protocol. Smoking status was classified into three groups: participants who had smoked over 100 cigarettes throughout their lifetimes but had quit before this study were ex-smokers, currently smoking were current smokers, and the remaining were non-smokers. Drinking status (alcohol intake) was also classified into three groups: current drinkers, ex-drinkers, and non-drinkers. Regularity of physical activity was determined according to whether subjects participated regularly in any sports to the point of sweating.

Body mass index (BMI) was calculated as weight in kilograms divided by height in meters squared (kg/m^2^). Systolic (SBP) and diastolic blood pressure (DBP) were measured twice by a standardized mercury sphygmomanometer (Baumanometer-Standby; W.A. Baum Co. Inc., New York, NY, USA). Blood samples were drawn after overnight fasting, and venous blood sampling was collected in a plain tube. Biochemical parameters, including fasting glucose, hemoglobin A1c (HbA1c), total cholesterol, HDL cholesterol, and triglycerides (TG), were determined by enzymatic methods (ADVIA 1650, Siemens, Tarrytown. NY, USA). LDL- cholesterol was calculated using the Friedewald equation (LDL-cholesterol = total cholesterol –HDL- cholesterol –[TG/5]).

### Definition of study phenotypes

HTN was defined as systolic BP ≥ 140 mmHg or diastolic BP ≥ 90 mmHg on health examination, currently taking an anti-hypertensive drug, or diagnosed by a physician. DM was defined as fasting blood glucose ≥ 126 mg/dl, HbA1c ≥ 6.5% (48 mmol/mol), currently taking an anti-diabetic drug or insulin, or diagnosed by a physician.

DL was defined as either diagnosis by a physician, current use of lipid-lowering medication, or according to the National Cholesterol Education Program- Adult Treatment Panel III (NCEP-ATP III) criteria: (1) hypercholesterolemia (serum TC ≥ 240 mg/dl), (2) hypertriglyceridemia (serum TG ≥ 200 mg/dl), (3) hyper-LDL-cholesterolemia (serum LDL-C ≥ 160 mg/dl), (4) hypo-HDL-cholesterolemia (serum HDL-C < 40 mg/dl).

### Outcome measurements

We defined CAD as the participant-reported history of the diagnosis or treatment of angina pectoris or myocardial infarction. Ischemic stroke (IS) was defined in the same manner, in that it was based on the participant-reported history of the diagnosis or treatment of ischemic stroke. Cardio-cerebrovascular disease (CCD) was defined as the combination of CAD and IS per our study outcome definition.

### Study design

**Fig. 1:**
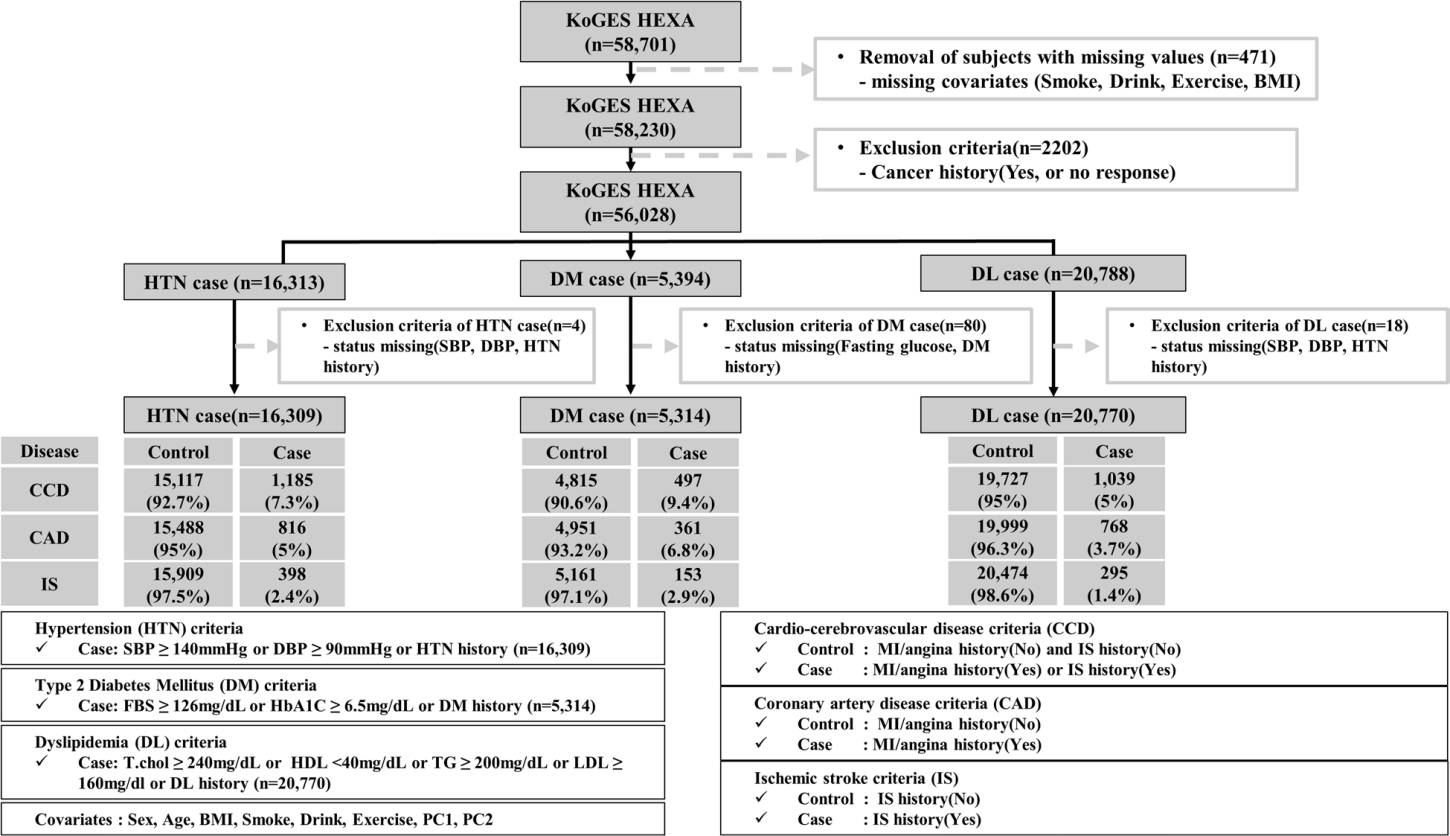
Study Design. Flow diagram illustrating the study sample selection process and overall study design

This study investigated the genetic risk factors of CVDs in the patients with metabolic disease (HTN, DM, or DL). For this analysis, we applied the exclusion criteria schematically illustrated in Fig. 1: from baseline we excluded participants with missing data values, i.e. smoking, alcohol, exercise history and body mass index (BMI) (*n* = 471). Subsequently, participants with histories of malignancy or no response regarding malignancy were excluded (*n* = 2,202). After these exclusions, 56,028 participants were included; the final sample size for the present analysis was 16,313 participants with HTN, 5394 participants with DM, and 20,788 participants with DL.

### Genotyping and quality control procedures

The genotype data were graciously provided by the Center for Genome Science, Korea National Institute of Health. The genotype data was produced by the Korea Biobank Array (Affymetrix, Santa Clara, CA, USA) (Moon et al., 2019, PMID 30718733). The experimental results of Korea Biobank Array were filtered by the quality control procedures of the following criteria: call rate higher than 97%, minor allele frequency higher than 1%, and Hardy–Weinberg equilibrium test p < 1 × 10^–5^. After the quality control procedures, the experimental genotypes were phased using ShapeIT v2 and IMPUTE v2 was used for imputation analysis of the phased genotype data with 1000 Genomes Phase3 data as a reference panel. After imputation, imputed variants with imputation quality score < 0.4 or MAF < 1% were excluded from further analysis (Moon et al., 2019, PMID 30718733). Finally, the number of SNPs for the GWAS was 7,975,321 SNPs from chromosomes 1 to 22. We associated the closest, or nearby genes of the significant variants as candidate genes utilizing LocusZoom.

### Statistical analysis

All data are presented as the mean ± standard deviation (SD), or number (%). For the discovery GWAS analysis, the association between individual SNP genotypes and CAD, IS, and CCD risks were modeled additively for each copy of the minor allele using logistic regression adjusted for age, sex, BMI, exercise status, smoking status, alcohol intake, and PC1 and PC2 as covariates using PLINK, version 1.9 [16]. PC1 and PC2 were obtained through a principal component analysis, which was conducted to reduce the bias of genomic data due to the regional differences in sample collection. We selected high linkage disequilibrium (LD) and cluster SNPs wherein no SNP gap exceeded 50 kb with high LD (r^2^ > 0.8) from the top significant SNPs. The significant associations were defined by genome-wide significance level *p*-values (5.00 × 10^–8^) [17]. The gene-region plot of the top SNP associations was generated with Locus- Zoom version 0.4.8.2 [18].

## Results

**Fig. 2:**
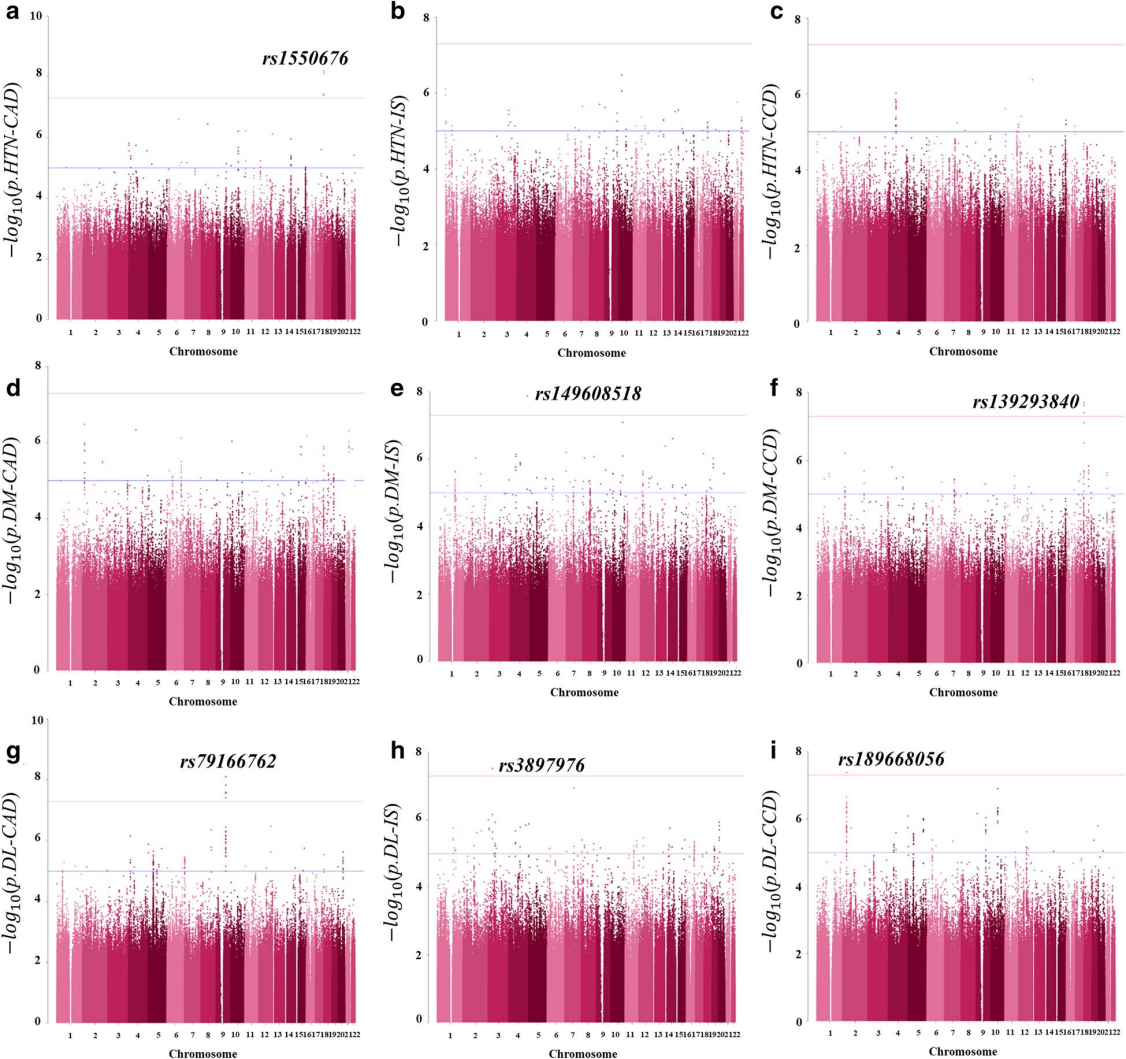
Manhattan Plots Showing Association Results of the GWASs. Manhattan plots showing association results from the GWASs of CVD in the HTN(A–C), DM(D–F), and DL(G–I) groups within the City Cohort: **a** HTN-CAD (816 CAD cases and 15,488 controls), **b** HTN-IS (398 IS cases and 15,909 controls), **c** HTN-CCD (1,185 CCD cases and 15,117 controls), **d** DM-CAD (361 CAD cases and 5,161 controls), **e** DM-IS (153 IS cases and 5,161 controls), **f** DM-CCD (497 CCD cases and 4,815 controls), **g** DL-CAD (768 CAD cases and 19,999 controls), **h** DL-IS (295 IS cases and 20,474 controls), and **i** DL-CCD (1,039 CCD cases and 19,727 controls). Each tested SNP is visualized as a dot with a location on the genome shown on the x-axis and log10- transformed p values on the y-axis. Results for all chromosomes within the consensus threshold for genome-wide significance (5 × 10^−8^) are marked with a horizontal line in red and suggestive significance (1 × 10^–5^) in blue

Clinical characteristics of the 42,393 individuals included in the GWAS are shown in Table 1, and further details categorized by disease status are in Additional file 1: Tables S1–S3*.* A total of 16,309 individuals with HTN (56.1% female and 57.28 [7.45] years old), 5,314 with DM (50.9% female and 57.79 [7.39] years old), and 20,770 with DL (58.4% female and 55.34 [7.63] years old) were included; the prevalence of CCD was 7.3% in HTN, 9.4% in DM, and 5% in DL, respectively. Results of the 9 GWASs are depicted in Fig. 2 as Manhattan plots using log10 transformed p-values. The leading SNPs in association with CAD, IS, and CCD for each group are described in Table 2. The full lists of genome-wide significant (*P*-value < 5 × 10^–8^) and suggestive (5 × 10^–8^ ≤ *P*-value < 1 × 10^–5^) SNPs from the GWASs are available in Additional file 2: Tables S4–S6.

**Table 1:** Clinical characteristics of the study population

Characteristics	HTN	DM	DL
Number of individuals, No.	16,309	5314	20,770
Female, No. (%)	9146 (56.1%)	2706 (50.9%)	12,125 (58.4%)
Age, y	57.28 (7.45)	57.79 (7.39)	55.34 (7.63)
Coronary artery disease, No. (%)	816 (5.0%)	361 (6.8%)	768 (3.7%)
Ischemic stroke, No. (%)	398 (2.4%)	153 (2.9%)	295 (1.4%)
Cardio-cerebrovascular disease, No. (%)	1185 (7.3%)	497 (9.4%)	1039 (5.0%)
Anthropometric traits			
Body mass index, kg/m^2^	25.04 (2.95)	25.12 (3.07)	24.68 (2.82)
Waist circumference, cm	84.37 (8.38)	85.52 (8.44)	83.37 (8.28)
Systolic blood pressure, mmHg	135.1 (14.58)	127.55 (14.93)	125.07 (14.64)
Diastolic blood pressure, mmHg	83.2 (9.88)	77.48 (9.37)	77.33 (9.61)
Biochemical traits			
Fasting plasma glucose, mg/dl	100.28 (23.37)	132.7 (38.71)	98.73 (23.12)
Total cholesterol, mg/dl	196.62 (36.79)	188.9 (40.78)	212.18 (43.92)
HDL cholesterol, mg/dl	51.65 (12.72)	49.01 (11.9)	48.44 (13.8)
Triglyceride, mg/dl	143.83 (95.78)	159.51 (119.82)	176.4 (114.14)
LDL cholesterol, mg/dl	116.23 (34.5)	108.02 (37.21)	128.47 (42.28)
Lifestyle factors			
Smoking status: Never/Quit/Current, No. (%)	10,979 (67.3%)/ 3497 (21.4%)/ 1833(11.2%)	3221 (60.6%)/ 1266 (23.8%)/ 827(15.6%)	13,889 (66.9%)/ 3849 (18.5%)/ 3032(14.6%)
Drinking status: Never/Quit/Current, No. (%)	7965 (48.8%)/ 760 (4.7%)/ 7584(46.5%)	2629 (49.5%)/ 325(6.1%)/ 2360(44.4%)	10,592 (51%)/ 891 (4.3%)/ 9287(44.7%)
Exercise status: No/Yes, No. (%)	7924 (48.6%)/ 8385 (51.4%)	2633 (49.5%)/ 2681(50.5%)	10,040 (48.3%)/ 10,730 (51.7%)

Continuous variables are presented as mean (SD). Categorical variables are presented as No. (%)

**Table 2:** Lead SNPs Associated with CVD at Genome-wide Significance by GWAS

	SNP	Position	Locus	Clustered SNPs	M/m	MAF	1000GenomeFreq Frequency			Genes	Feature	CAD		IS		CCD	
							Asian	Eur	Amer			OR	P	OR	P	OR	P
HTN	rs1550676	17:77,801,055	17q25.3	rs74608211, rs145216691, rs559385953	T/C	0.01176	–	–	–	CBX8-CBX4, CBX2 GAA CARD14 SLC26A11	Intergenic	2.607 (1.886–3.602)	6.37E−09	0.5929 (0.2625–1.339)	2.09E−01	1.927 (1.417–2.621)	2.90E−05
DM	rs149608518	4:165,472,675	4q32.3	–	C/T	0.01035	0.0079	0	0	MARCH1-LINC01207, APELA	Intergenic	1.425 (0.7313–2.775)	2.98E−01	5.587 (3.086–10.12)	1.34E−08	2.68 (1.639–4.384)	8.55E−05
	rs139293840	17:78,530,359	17q25.3	rs957271283, rs147509862	G/A	0.01013	0.0089	0.001	0	RPTOR, RNF213 CARD14 SLC26A11	Intron	3.359 (2.058–5.484)	1.27E−06	3.111 (1.581–6.124)	1.02E−03	3.511 (2.265–5.444)	1.99E−08
DL	rs189668056	2:38,308,827	2p22.2	rs189258819	G/A	0.02031	0.0129	0	0.0187	CYP1B1- CYP1B1-AS1	Intergenic	1.942 (1.453–2.596)	7.30E−06	2.147 (1.401–3.292)	4.55E−04	2.027 (1.574–2.609)	4.24E−08
	rs3897976	3:41,606,692	3p22.1	–	G/A	0.05095	0.0823	0.1074	0.1153	ULK4, CTNNB1	Intron	1.008 (0.801–1.269)	9.46E−01	2.162 (1.646–2.84)	2.97E−08	1.301 (1.083–1.562)	4.86E−03
	rs79166762	9:92,466,006	9q22.2	rs12115796, rs12115684, rs12115802, rs75847266, rs117445944, rs75273685, rs76097735, rs78819980, rs79641632	T/C	0.01918	0.0159	0	0	UNQ6494- LOC101927847, GADD45G SEMA4D	Intergenic	2.282 (1.724–3.019)	7.78E−09	1.207 (0.6891–2.114)	5.11E−01	1.928 (1.483–2.505)	9.17E−07

Lead SNPs were selected within genome-wide significant variants by lowest p-values. Genes include positional candidate genes within 400 kb of SNP, and initial mapped gene or genetic region is listed first before a comma. Odds ratios are shown as odds ratio (95% confidence interval). SNP, single nucleotide polymorphism; M/m, Major/minor allele; MAF, minor allele frequency; OR, odds ratio; P, p-value

### GWAS results of CVDs in Hypertension

The GWAS of HTN-CAD (Fig. 2a) showed 1 genome-wide significant association locus (17q25.3) and 19 loci of suggestive association, HTN- IS (Fig. 2b) showed 23 suggestive loci, and HTN-CCD (Fig. 2c) showed 10 suggestive loci. The leading SNP of CAD in HTN (17q25.3/*CBX8-CBX4* rs1550676) showed the strongest association (*P* = 6.37 × 10^−9^) and a high odds ratio (OR) of 2.607 (95% CI 1.886–3.602), and a total of 4 significant SNPs were clustered in the locus.

### GWAS results of CVDs in DM

The GWAS of DM-CAD (Fig. 2d) showed 18 suggestive loci and that of DM-IS (Fig. 2e) showed 1 novel significant locus (4q32.2) and 41 suggestive loci. In the GWAS of DM-CCD (Fig. 2f), 1 locus, 17q25.3, was again shown to be significant with 21 suggestive loci. The leading SNP of CAD and CCD in DM (17q25.3/*RPTOR* rs139293840) showed strong associations (*P* = 1.99 × 10^−8^) as well as a high OR (3.511, 95%CI 2.265–5.444). The leading SNP for IS (4q32.3/*MARCH1-LINC01207* rs149608518) also showed significance (*P* = 1.34 × 10^−8^) and a significantly high OR (5.587, 95%CI 3.086–10.12).

### GWAS results of CVDs in DL

In DL, all 3 GWASs identified genome-wide significant loci: in DL-CAD (Fig. 2g), 1 significant locus (9q22.2) and 24 suggestive loci; in DL-IS (Fig. 2h), 1 significant locus (3p22.1) and 40 suggestive loci; and in DL-CCD (Fig. 2i), 1 significant locus (2p22.2) and 19 suggestive loci. A total of 11 significant SNPs were clustered in 9q22.2/*UNQ6494-LOC10192784* associated with CAD, the lead SNP being rs79166762 (*P* = 7.78 × 10^−9^, OR = 2.282 [95% CI 1.724–3.019]). 3p22.1/*ULK4* rs3897976 showed significance for IS (*P* = 2.97 × 10^−8^, OR = 2.162 [95% CI 1.646–2.84]) and 2p22.2/*CYP1B1*-*CYP1B1-AS1* rs189668056 for CCD (*P* = 4.24 × 10^−8^, OR = 2.027 [95% CI 1.574–2.609]).

## Discussion

Despite large-scale GWASs providing robust evidence for genetic variants influencing metabolic pathways and CVDs, [19–24] it is unclear whether genetic variants exist for CVD within the basis of individual chronic metabolic diseases. The aim of our study was to identify genetic variations associated with CVD among individuals with HTN, DM, and DL, respectively; for which we found varied meaningful loci for CVD.

In HTN, locus 17q25.3 [*CBX8-CBX4*] showed the highest significance for CAD; intriguingly, 17q25.3 was also found to uniformly show suggestive associations for CAD in both DM and DL, which shall be discussed in detail later. Candidate genes for HTN-CAD risk include *CBX2/4/8, GAA, CARD14*, and *SLC26A11*. The *CBX* gene family, a recent research interest in the field of long noncoding RNAs, has been associated with CVD and atherosclerosis in multiple studies [25, 26]. *GAA* gene expression has been found to be specifically increased in CAD patients than in controls [27]. *CARD14*, which is mainly associated with psoriasis (ongoing clinical studies suggest increased risk of CVDs in psoriasis) and auto-inflammatory disorders, acts as a pro-inflammatory gene by affecting *NF-kB* activation in the IL-17 inflammation pathway. Puig et al. [28] found that expression of genes such as *CARD14* classified human atherosclerotic plaque by relative inflammation status. *SLC26A11*, a product of the *SLC26A* family of anion transporters, has been associated with both cardiovascular and cerebrovascular disease; notably, it was postulated that *SLC26A11* tagged multiple variants of *RNF213* associated with young-onset IS [29], and *SLC26A11* was detected from blood samples of patients with ST-elevation MI [30].

Interestingly, *CARD14* and *SLC26A11* in the region of 17q25.3 were located within 400 kb of the lead SNP for CCD in DM (17q25.3*/RPTOR* rs139293840) as well. HTN and DM share common risk factors and frequently co-occur [31]; additionally, CVD is the most common long-term complication of both [6]. The overlapping association of SNPs and genetic loci between HTN and DM support the existence of shared points of regulation for these phenotypes [31].

*RPTOR, RNF213, CARD14*, and *SLC26A11* all bear the potential to be the mechanism behind DM-CCD risk loci 17q25.3 [*RPTOR*]. *RNF213* plays important roles in vascular development, and mutant *RNF213* has been shown to reduce angiogenesis and induce endothelial dysfunction [32]. *RNF213* has been identified as a susceptibility gene of Moyamoya disease [33], intracranial arterial stenoses [34], and systemic vasculopathy among East Asian populations [33] and CAD in the Japanese population [34]. Recently, the significance of *RNF213* and *SLC26A11* in Caucasian populations were reported as well [29], which encourages its re-evaluation within other ethnicities. *RPTOR* is a component of the *mTOR* pathway, which regulates cell growth in response to nutrient levels by associating with the mammalian target of rapamycin (*mTOR*) [35]. New insights have been offered into *RPTOR* and CVD/atherosclerosis; such as evidence that miR-100 exerts anti-angiogenic properties through suppression of *mTOR*, [25] and that inhibition of cardiac *GSK-3β* during continued myocardial ischemia attenuates ischemia-induced *mTORC1* inhibition and increases ischemic injury [35].

Rs139293840 (17q25.3/*RPTOR*, minor allele frequency (MAF) ~ 0.01) showed substantial risk for DM-CAD (OR 3.36 [2.06–5.48]); although the association was suggestive (*P* = 1.27 × 10^−6^; however, genome-wide significance in DM-CCD, *P* = 1.99 × 10^–8^), this could be due to insufficient statistical power after selecting populations for quality control. Genetic risk factors regarding CAD have usually been found to have only a modest effect, conferring an OR ranging from 1.05 to 1.20 [36]; considering the effect size, further replication studies with larger sample sizes are warranted. Based on genotyping data, the MAF of rs139293840 varies among different ethnic populations, ranging from 0.89% in Asians to 0.01% in the population of European ancestry to 0% in the American population. Thus, the association between rs139293840 and CAD may be more prevalent in the Asian population.

The locus, 4p32.3 [*MARCH1-LINC01207*] showed significant risk for DM-IS (OR 5.587 [3.086–10.12]) and was located near *APELA* and *MARCH1*. *APELA* is a novel endogenous peptide ligand that activates the APJ receptor axis and is cardioprotective against HTN, MI, pulmonary arterial hypertension, and heart failure [37, 38]. Recently, studies have shown that the apelin/APJ axis possess neuroprotective effects by inhibiting neuronal apoptosis and improving functional recovery in IS through diverse mechanisms, including suppressing inflammatory responses, modulating autophagy, and promoting angiogenesis [39]. *MARCH1* has been identified as a susceptibility locus for MI and HTN in Japanese [40], and regarding IS, was included as 1 of the 31 SNPs significantly associated with IS in an initial EWAS [41].

In DL-CAD risk, locus 9q22.2 [*UNQ6494-LOC10192784*] was significant; Semaphorin 4D (*SEMA4D*) and Growth arrest and DNA-damage-inducible 45 (*GADD45G*) are potential candidates. *Sema4D* is a type I integral membrane glycoprotein expressed by most hematopoietic cells that participate in the pathogenesis of atherosclerosis, which has been identified as an independent risk factor for CHD [42]. *GADD45G,* of the *GADD45* family involved in p38 mitogen-activated protein kinase-dependent cell death, has been linked with several cardiologic studies [43]. Recently, conferred resistance to myocardial ischemic injury and cardiomyocyte apoptosis was found when *GADD45G* was deleted in mice [44].

Again, the gene-rich locus 17q25.3 was found to be of suggestive association for CAD in DL. Considering the small sample size, we consider this finding to bear significance, which confers on 17q25.3 the status of a common susceptibility locus of CVD in HTN, DM, and DL. It has not been fully elucidated whether these chronic metabolic diseases are part of the causal pathway for CVD or merely comorbid diseases in “common soil”. The search has been fraught with controversial findings, as has the presence of overlap in the genetic architecture of metabolic diseases and CVDs. This study provides first probable evidence of a common candidate locus for CVD in the 3 diseases using a GWAS approach.

Locus 3p22.1 [*ULK4*] of DL-IS risk seems likely to be associated with *ULK4*; recently, *ULK4* was identified by GWAS and brain eQTL to be a susceptibility gene for IS and small vessel stroke in trans-ethnic datasets [45]. However, *CTNN1B* of the *WNT* gene family is another possibility. The Wnt signaling pathway contributes to the development of CVDs; both canonical and noncanonical Wnt signaling cascades moderate cell phenotypic modulation of vascular smooth muscles in CVDs [46]. *CTNN1B* encodes beta-catenin and is essential in maintaining CNS homeostasis [47]; in terms of arteriosclerotic effects, *CTNN1B* was found to harbor a susceptibility variant for MI in the Han population [48]. Recently, expression of vital genes in the Wnt pathway (including *CTNN1B*) was found to be upregulated in cerebral infarction in rats [46]; it seems plausible that similar findings could be found in humans.

Finally, 2p22.2 [*CYP1B1-CYP1B1-AS1*] of DL-CCD risk could be associated with Cytochrome P450 1B1(*CYP1B1*), a member of the well-known Cytochrome P450 family. *CYP1B1* is expressed in various tissues, with the highest mRNA expression levels found extrahepatically, such as the heart and brain [49]. Recent findings have shown that *CYP1B1* contributes to the development of atherosclerosis, DL, and HTN in rats [50]; demonstrates an association with DM [51]; and has a suggested influence on adipogenesis [49].

### Strengths and limitations

There are limitations to our study. First, replication is lacking, and the findings may not be generalized to cohorts with different ethnicities and races. Second, we investigated the phenotypes of diseases using a patient-reported questionnaire and thus could not investigate various CVDs or measurable continuous traits. Third, this work is based on statistical evidence and does not provide functional experimental validation. Potential genetic interactions using biological and mechanical analyses are required to confirm these findings, and additional studies are warranted for replication and validation.

Despite these limitations, our study has several unique strengths. Many studies have demonstrated varied findings regarding the genetic relations between CVD and its risk factors [52–54]. However, most previous studies utilized variants of genome-wide significance or genotyping arrays that focused only on pre-selected loci, and they have not fully utilized genome-wide variation for the estimation of genetic variants of CVD risk [55–57]. Moreover, none of these previous studies have focused on the genetic variants of CVD within the same chronic conditions. To the best of our knowledge, the present study provides novel evidence of genetic associations with CVD specific to individuals with HTN, DM, and DL. Charmet et al. [58] found novel candidates for CAD using a then unprecedented similar study design (434 cases and 3,123 controls), in patients with type 1 diabetes. The barrier of limited statistical power due to sample size may be why such studies are lacking.

Although some of our findings showed low prevalence, the overall effect size is significant with high ORs. Also, the allele frequency may be underestimated due to smaller quality genomic databases of East Asians used in forming the reference panels. Further, cardio-metabolic diseases associated with CVDs have been increasing in prevalence as well as in total medical costs in Asians, with HTN and DM ranking 1st and 2nd among all medical diagnoses in Korea, respectively [59]. Dyslipidemia prevalence has been rapidly increasing up to 38.4% of all adults, and CVDs account for 1/4 of total mortality of the Korean population according to recent data [60]. Our preliminary findings may be used to improve the identification of individuals with HTN, DM, or DL at high-risk for cardiovascular events or to provide novel targets for potential pharmacologic development. In the era of personalized, preventive medicine with progressively aging populations with chronic cardio-metabolic diseases, further comprehensive analyses of our findings seem highly warranted.

## Conclusions

In conclusion, our GWAS, of unprecedented design, provides several novel insights into the genetic architecture of CVDs. We report 6 loci with genome-wide significant association for CVDs, which harbor numerous potential positional candidate genes, and show substantial CVD risk discrimination in individuals with HTN, DM, or DL. In addition, we suggest the importance of 17q25.3 as a potential common CAD locus for HTN, DM, and DL.

## Supplementary Information

**Additional file 1: Table S1.** Baseline Characteristics of the Hypertension Study Population. **Table S2.** Baseline Characteristics of the Diabetes Mellitus Study Population. **Table S3.** Baseline Characteristics of the Dyslipidemia Study Population. **Figure S1.** Quantile-quantile plots with genomic inflation values**. Figure S2.** Regional association plots for lead SNPs, **A **HTN-CAD **B **DM-IS **C **DM-CCD **D **DL-CAD **E **DL-CCD **F **DL-IS.

**Additional file 2: Table S4. **Original GWAS results from discovery for association with CVD in HTN.** Table S5. **Original GWAS results from discovery for association with CVD in DM. **Table S6. **Original GWAS results from discovery for association with CVD in DL.

## Data Availability

The datasets used and/or analyzed during the current study are available from the corresponding authors on reasonable request.

## Abbreviations

BMI: Body mass index
CAD: Coronary artery disease
IS: Ischemic stroke
CVD: Cardiovascular disease
GWAS: Genome-wide association study
HDL: High-density lipoprotein
LDL: Low-density lipoprotein
SBP: Systolic blood pressure
SNP: Single-nucleotide polymorphism
DM: Type 2 diabetes mellitus
DL: Dyslipidemia
KoGES: Korean Genome and Epidemiology Study
NCEP-ATP III: National Cholesterol Education Program- Adult Treatment Panel III
PAH: Pulmonary artery hypertension
HF: Heart failure
