# Supplementary material for: Identification of susceptibility loci for cardiovascular disease in adults with hypertension, diabetes, and dyslipidemia
Source: J Transl Med. 2021 Feb 25;19:85. doi: 10.1186/s12967-021-02751-3 (PMC7905883; doi:10.1186/s12967-021-02751-3)

**Supplementary Information**

**Identification of Susceptibility Loci for Cardiovascular Disease in Adults with Hypertension, Diabetes, and Dyslipidemia**

Short title: Novel genetic approach of cardiovascular disease

Youhyun Song, MD^1,†^, Ja-Eun Choi, BS^2,†^, Yu-Jin Kwon, MD^3^, Hyuk-Jae Chang, MD, PhD^4^, Jung Oh Kim, PhD^2^, Da-Hyun Park, BS^2^, Jae-Min Park, MD^1^, Seong-Jin Kim, PhD^2^, Ji Won Lee, MD, PhD^1,*^, and Kyung-Won Hong, PhD^2,*^

^1^ Department of Family Medicine, Gangnam Severance Hospital, Yonsei University College of Medicine, 211, Eonju‐ro, Gangnam‐gu, Seoul 06273, Korea

^2^ Theragen Bio Co., Ltd., Suwon, Gyeonggi-do, 16229, Republic of Korea

^3^ Department of Family Medicine, Yongin Severance Hospital, Yonsei University College of Medicine, 363, Dongbaekjukjeon-daero, Giheung-gu, Yongin-si 16995, Gyeonggi-do, Korea

^4^ Division of Cardiology, Severance Cardiovascular Hospital, Yonsei University College of Medicine, 50-1, Yonsei-ro, Seodaemun-gu, Seoul 03722, Korea

† These authors are co‐first authors who equally contributed to this work.

* Corresponding authors

**Table S1.** Baseline Characteristics of the Hypertension Study Population

**Table S2.** Baseline Characteristics of the Diabetes Mellitus Study Population

**Table S3.** Baseline Characteristics of the Dyslipidemia Study Population

**Table S4**. Original GWAS results from discovery for association with CVD in HTN

**Table S5.** Original GWAS results from discovery for association with CVD in DM

**Table S6.** Original GWAS results from discovery for association with CVD in DL

**Figure S1.** Quantile-quantile plots with genomic inflation values

**Figure S2**. Regional association plots for lead SNPs

**A)**HTN-CAD **B)**DM-IS **C)**DM-CCD **D)**DL-CAD **E)**DL-CCD **F)**DL-IS

**cf) Tables S4-6** are available separately in ***Additional file 2 (.xls)***.

| **Table S1. Baseline Characteristics of the Hypertension Study Population** | | | | | | |
| --- | --- | --- | --- | --- | --- | --- |
| **Characteristics** | **Cardio-cerebrovascular Disease** | | **Coronary Artery Disease** | | **Ischemic Stroke** | |
|  | **case** | **control** | **case** | **control** | **case** | **control** |
| Number of individuals | 1185(7.3%) | 15117(92.7%) | 816(5%) | 15488(95%) | 398(2.4%) | 15909(97.5%) |
| Gender: Female, n (%) | 549(46.3%) | 8592(56.8%) | 385(47.2%) | 8758(56.5%) | 175(44%) | 8969(56.4%) |
| Age, y | 60.96±6.37 | 56.99±7.45 | 61.2±6.17 | 57.07±7.45 | 60.38±6.71 | 57.2±7.45 |
| CCD, n (%) | 1185(100%) | 0(0%) | 816(100%) | 369(2.4%) | 398(100%) | 787(4.9%) |
| CAD, n (%) | 816(68.9%) | 0(0%) | 816(100%) | 0(0%) | 29(7.3%) | 787(4.9%) |
| IS, n (%) | 398(33.6%) | 0(0%) | 29(3.6%) | 369(2.4%) | 398(100%) | 0(0%) |
| DM, n (%) | 321(27.1%) | 2472(16.4%) | 225(27.6%) | 2568(16.6%) | 109(27.4%) | 2685(16.9%) |
| DL, n (%) | 626(52.8%) | 7178(47.5%) | 452(55.4%) | 7352(47.5%) | 193(48.5%) | 7613(47.9%) |
| HTN and DM and DL, n (%) | 193(16.3%) | 1474(9.8%) | 134(16.4%) | 1533(9.9%) | 67(16.8%) | 1600(10.1%) |
| **Anthropometric traits** | | | | | | |
| Body mass index, kg/m2 | 25.5±2.9 | 25.4±2.9 | 25.6±3.1 | 25.4±2.9 | 25.1±2.5 | 25.4±2.9 |
| Waist circumference, cm | 86.4±8.1 | 85.6±8.2 | 86.6±8.3 | 85.6±8.2 | 85.9±7.5 | 85.6±8.2 |
| Systolic blood pressure, mmHg | 131.3±14.8 | 135.3±14.6 | 130.8±14.9 | 135.2±14.6 | 132.5±14.8 | 135±14.7 |
| Diastolic blood pressure, mmHg | 79.2±9.7 | 83.3±9.8 | 79.2±10 | 83.2±9.8 | 79.5±8.9 | 83.1±9.9 |
| **Biochemical traits** | | | | | | |
| Fasting plasma glucose, mg/dl | 103.6±26 | 102.7±25.6 | 103.4±26.3 | 102.8±25.6 | 104.2±25.7 | 102.8±25.6 |
| Total cholesterol, mg/dl | 175.8±41.6 | 207.3±43 | 173.1±41.7 | 206.7±43.1 | 181.7±39.7 | 205.4±43.7 |
| HDL cholesterol, mg/dl | 44.7±11.4 | 48.1±13.2 | 44.6±11.3 | 48±13.1 | 44.5±11.2 | 47.9±13.1 |
| Triglyceride, mg/dl | 165.9±99 | 187.6±119.7 | 163.1±104.8 | 187.3±118.9 | 172.5±82 | 186.3±119 |
| LDL cholesterol, mg/dl | 97.9±37.6 | 121.7±42.1 | 95.9±38 | 121.3±42 | 102.6±35.5 | 120.2±42.3 |
| **Lifestyle factors** | | | | | | |
| Smoking status: Never/Quit/Current, n (%) | 687(58%) / 368(31.1%) / 130(11%) | 10287(68%) / 3127(20.7%) / 1703(11.3%) | 484(59.3%) / 238(29.2%) / 94(11.5%) | 10492(67.7%) / 3257(21%) / 1739(11.2%) | 219(55%) / 139(34.9%) / 40(10.1%) | 10758(67.6%) / 3358(21.1%) / 1793(11.3%) |
| Drinking status: Never/Quit/Current, n (%) | 598(50.5%) / 135(11.4%) / 452(38.1%) | 7363(48.7%) / 625(4.1%) / 7129(47.2%) | 425(52.1%) / 76(9.3%) / 315(38.6%) | 7538(48.7%) / 684(4.4%) / 7266(46.9%) | 190(47.7%) / 64(16.1%) / 144(36.2%) | 7773(48.9%) / 696(4.4%) / 7440(46.8%) |
| Exercise status: No/Yes, n (%) | 604(51%) / 581(49%) | 7316(48.4%) / 7801(51.6%) | 410(50.2%) / 406(49.8%) | 7511(48.5%) / 7977(51.5%) | 212(53.3%) / 186(46.7%) | 7711(48.5%) / 8198(51.5%) |

Continuous variables are presented as mean±SD. Categorical variables are presented as n (%).

| **Table S2. Baseline Characteristics of the Diabetes Mellitus Study Population** | | | | | | |
| --- | --- | --- | --- | --- | --- | --- |
| **Characteristics** | **Cardio-cerebrovascular Disease** | | **Coronary Artery Disease** | | **Ischemic Stroke** | |
|  | **case** | **control** | **case** | **control** | **case** | **control** |
| Number of individuals | 497(9.4%) | 4815(90.6%) | 361(6.8%) | 4951(93.2%) | 153(2.9%) | 5161(97.1%) |
| Gender: Female, n (%) | 194(39%) | 2511(52.1%) | 136(37.7%) | 2569(51.9%) | 62(40.5%) | 2644(51.2%) |
| Age, y | 61.52±5.92 | 57.39±7.41 | 61.48±5.98 | 57.51±7.4 | 61.63±5.77 | 57.67±7.39 |
| CCD, n (%) | 497(100%) | 0(0%) | 361(100%) | 136(2.7%) | 153(100%) | 344(6.7%) |
| CAD, n (%) | 361(72.6%) | 0(0%) | 361(100%) | 0(0%) | 17(11.1%) | 344(6.7%) |
| IS, n (%) | 153(30.8%) | 0(0%) | 17(4.7%) | 136(2.7%) | 153(100%) | 0(0%) |
| HTN, n (%) | 321(64.6%) | 2472(51.3%) | 225(62.3%) | 2568(51.9%) | 109(71.2%) | 2685(52%) |
| DL, n (%) | 281(56.5%) | 2631(54.6%) | 202(56%) | 2710(54.7%) | 89(58.2%) | 2823(54.7%) |
| HTN and DM and DL, n (%) | 193(38.8%) | 1474(30.6%) | 134(37.1%) | 1533(31%) | 67(43.8%) | 1600(31%) |
| **Anthropometric traits** | | | | | | |
| Body mass index, kg/m2 | 25.5±3 | 25.5±3 | 25.7±3.1 | 25.5±3 | 25.3±2.6 | 25.5±3 |
| Waist circumference, cm | 87.4±8.4 | 86.6±8.2 | 87.9±8.6 | 86.6±8.2 | 86.5±7.8 | 86.7±8.2 |
| Systolic blood pressure, mmHg | 127.8±16.1 | 128.6±14.7 | 127.1±16.2 | 128.7±14.8 | 130.6±16.1 | 128.5±14.8 |
| Diastolic blood pressure, mmHg | 76.4±9.9 | 78.2±9.2 | 76.3±9.8 | 78.2±9.2 | 77±10.2 | 78.1±9.2 |
| **Biochemical traits** | | | | | | |
| Fasting plasma glucose, mg/dl | 126±35.2 | 135.4±40.6 | 127±37 | 135.1±40.4 | 122.9±30.5 | 134.9±40.4 |
| Total cholesterol, mg/dl | 164.6±41.4 | 199.2±46.4 | 162.7±39.9 | 198.3±46.6 | 169.2±44 | 196.7±46.9 |
| HDL cholesterol, mg/dl | 42.8±11.5 | 45.8±11.9 | 42.3±11.8 | 45.7±11.9 | 44.1±10.8 | 45.5±11.9 |
| Triglyceride, mg/dl | 171.6±107.8 | 205±147.4 | 176.8±119.5 | 203.6±146 | 158.2±69.8 | 203.1±146 |
| LDL cholesterol, mg/dl | 87.5±38 | 112.4±43.7 | 85.1±37.2 | 111.8±43.7 | 93.5±38 | 110.5±43.9 |
| **Lifestyle factors** | | | | | | |
| Smoking status:  Never/Quit/Current, n (%) | 253(50.9%) / 170(34.2%) / 74(14.9%) | 2967(61.6%) / 1095(22.7%) / 753(15.6%) | 180(49.9%) / 123(34.1%) / 58(16.1%) | 3040(61.4%) / 1142(23.1%) / 769(15.5%) | 81(52.9%) / 54(35.3%) / 18(11.8%) | 3140(60.8%) / 1212(23.5%) / 809(15.7%) |
| Drinking status:  Never/Quit/Current, n (%) | 247(49.7%) / 52(10.5%) / 198(39.8%) | 2380(49.4%) / 273(5.7%) / 2162(44.9%) | 183(50.7%) / 31(8.6%) / 147(40.7%) | 2444(49.4%) / 294(5.9%) / 2213(44.7%) | 73(47.7%) / 23(15%) / 57(37.3%) | 2556(49.5%) / 302(5.9%) / 2303(44.6%) |
| Exercise status: No/Yes, n (%) | 248(49.9%) / 249(50.1%) | 2385(49.5%) / 2430(50.5%) | 177(49%) / 184(51%) | 2456(49.6%) / 2495(50.4%) | 79(51.6%) / 74(48.4%) | 2554(49.5%) / 2607(50.5%) |

Continuous variables are presented as mean±SD. Categorical variables are presented as n (%).

| **Table S3. Baseline Characteristics of the Dyslipidemia Study Population** | | | | | | |
| --- | --- | --- | --- | --- | --- | --- |
| **Characteristics** | **Cardio-cerebrovascular Disease** | | **Coronary Artery Disease** | | **Ischemic Stroke** | |
|  | **case** | **control** | **case** | **control** | **case** | **control** |
| Number of individuals | 1039(5%) | 19727(95%) | 768(3.7%) | 19999(96.3%) | 295(1.4%) | 20474(98.6%) |
| Gender: Female, n (%) | 471(45.3%) | 11651(59.1%) | 357(46.5%) | 11766(58.8%) | 124(42%) | 12000(58.6%) |
| Age, y | 60.3±6.41 | 55.07±7.59 | 60.32±6.36 | 55.14±7.6 | 60.21±6.45 | 55.27±7.62 |
| CCD, n (%) | 1039(100%) | 0(0%) | 768(100%) | 271(1.4%) | 295(100%) | 744(3.6%) |
| CAD, n (%) | 768(73.9%) | 0(0%) | 768(100%) | 0(0%) | 24(8.1%) | 744(3.6%) |
| IS, n (%) | 295(28.4%) | 0(0%) | 24(3.1%) | 271(1.4%) | 295(100%) | 0(0%) |
| HTN, n (%) | 626(60.3%) | 7178(36.4%) | 452(58.9%) | 7352(36.8%) | 193(65.4%) | 7613(37.2%) |
| DM, n (%) | 281(27%) | 2631(13.3%) | 202(26.3%) | 2710(13.6%) | 89(30.2%) | 2823(13.8%) |
| HTN and DM and DL, n (%) | 193(18.6%) | 1474(7.5%) | 134(17.4%) | 1533(7.7%) | 67(22.7%) | 1600(7.8%) |
| **Anthropometric traits** | | | | | | |
| Body mass index, kg/m2 | 25.1±2.8 | 24.6±2.8 | 25.2±2.9 | 24.6±2.8 | 24.9±2.5 | 24.7±2.8 |
| Waist circumference, cm | 85.8±7.9 | 83.2±8.3 | 86±8.1 | 83.3±8.3 | 85.5±7.5 | 83.3±8.3 |
| Systolic blood pressure, mmHg | 126.3±14.6 | 125±14.6 | 125.7±14.6 | 125±14.6 | 128.1±14.8 | 125±14.6 |
| Diastolic blood pressure, mmHg | 76.7±9.4 | 77.4±9.6 | 76.6±9.6 | 77.4±9.6 | 77.1±8.9 | 77.3±9.6 |
| **Biochemical traits** | | | | | | |
| Fasting plasma glucose, mg/dl | 102.2±25.1 | 98.5±23 | 102.4±25.7 | 98.6±23 | 102±23.7 | 98.7±23.1 |
| Total cholesterol, mg/dl | 178.7±43.3 | 213.9±43.2 | 176±42.9 | 213.6±43.4 | 184.3±43.2 | 212.6±43.8 |
| HDL cholesterol, mg/dl | 44.4±11.7 | 48.6±13.9 | 44.3±11.6 | 48.6±13.8 | 44.7±11.7 | 48.5±13.8 |
| Triglyceride, mg/dl | 163±93.1 | 177.1±115.1 | 161.3±97.8 | 177±114.7 | 167.2±78.7 | 176.5±114.6 |
| LDL cholesterol, mg/dl | 101.6±39.7 | 129.9±41.9 | 99.5±39.7 | 129.6±42 | 106.2±39 | 128.8±42.2 |
| **Lifestyle factors** | | | | | | |
| Smoking status: Never/Quit/Current, n (%) | 581(55.9%) / 317(30.5%) / 141(13.6%) | 13305(67.4%) / 3531(17.9%) / 2891(14.7%) | 440(57.3%) / 225(29.3%) / 103(13.4%) | 13447(67.2%) / 3623(18.1%) / 2929(14.6%) | 155(52.5%) / 101(34.2%) / 39(13.2%) | 13733(67.1%) / 3748(18.3%) / 2993(14.6%) |
| Drinking status: Never/Quit/Current, n (%) | 543(52.3%) / 127(12.2%) / 369(35.5%) | 10046(50.9%) / 764(3.9%) / 8917(45.2%) | 420(54.7%) / 82(10.7%) / 266(34.6%) | 10170(50.9%) / 809(4%) / 9020(45.1%) | 138(46.8%) / 50(16.9%) / 107(36.3%) | 10453(51.1%) / 841(4.1%) / 9180(44.8%) |
| Exercise status:  No/Yes, n (%) | 491(47.3%) / 548(52.7%) | 9548(48.4%) / 10179(51.6%) | 358(46.6%) / 410(53.4%) | 9681(48.4%) / 10318(51.6%) | 145(49.2%) / 150(50.8%) | 9895(48.3%) / 10579(51.7%) |

Continuous variables are presented as mean±SD. Categorical variables are presented as n (%).

**Tables S4-6.** Spreadsheets of original GWAS results from discovery, see ***Additional file 2 (.xls).***

**Figure S1.** Quantile-quantile plots with genomic inflation values


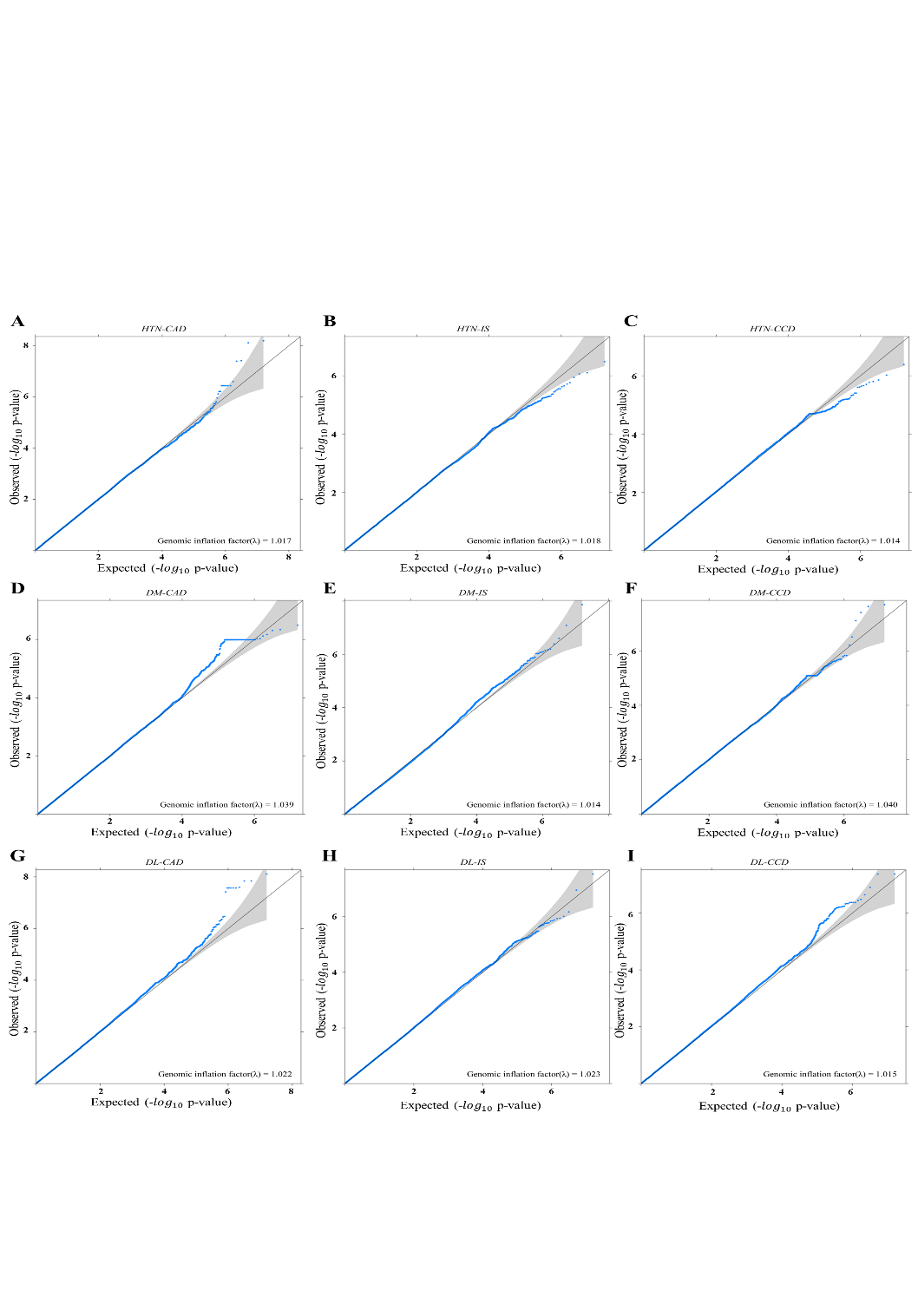


**Figure S2**. Regional association plots for lead SNPs

**A)**HTN-CAD **B)**DM-IS **C)**DM-CCD **D)**DL-CAD **E)**DL-CCD **F)**DL-IS

1. HTN-CAD


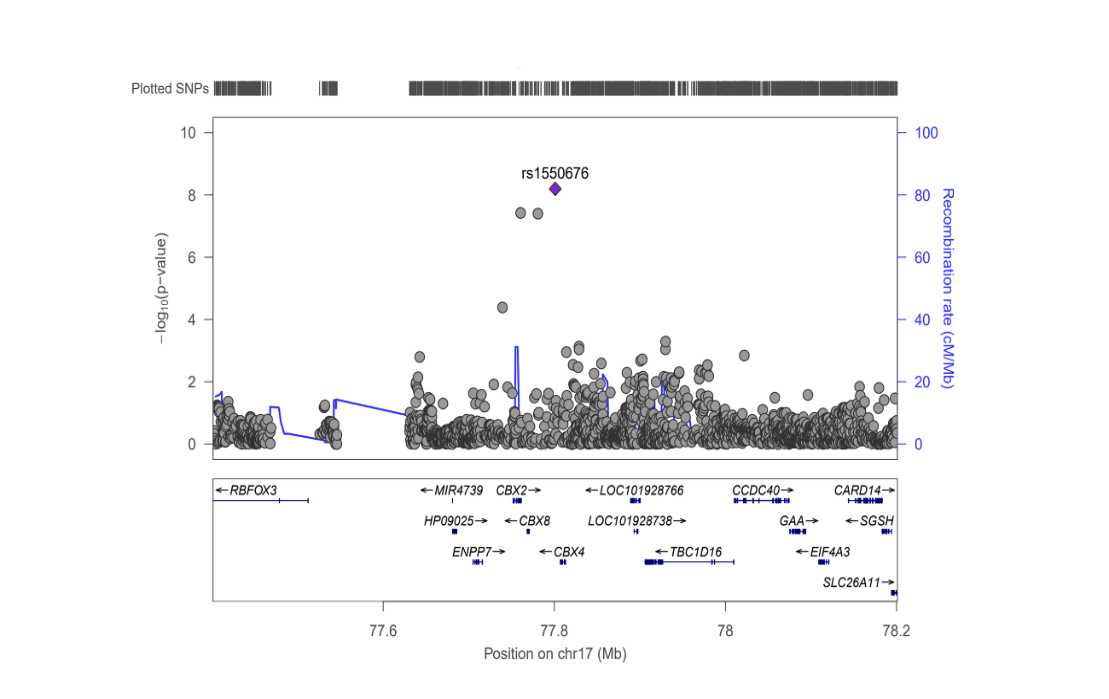


Cf) There is no result of LD analysis about the snp "rs1550676" in 1000Genome reference panel.

1. DM-IS


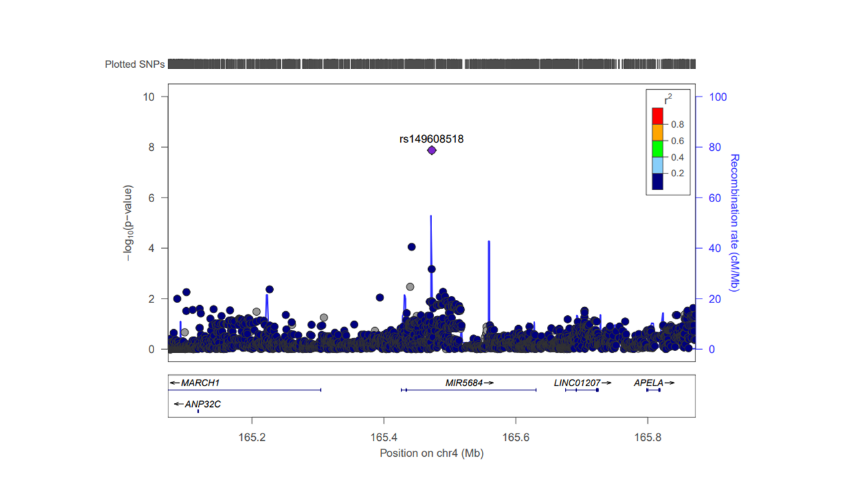


1. DM-CCD


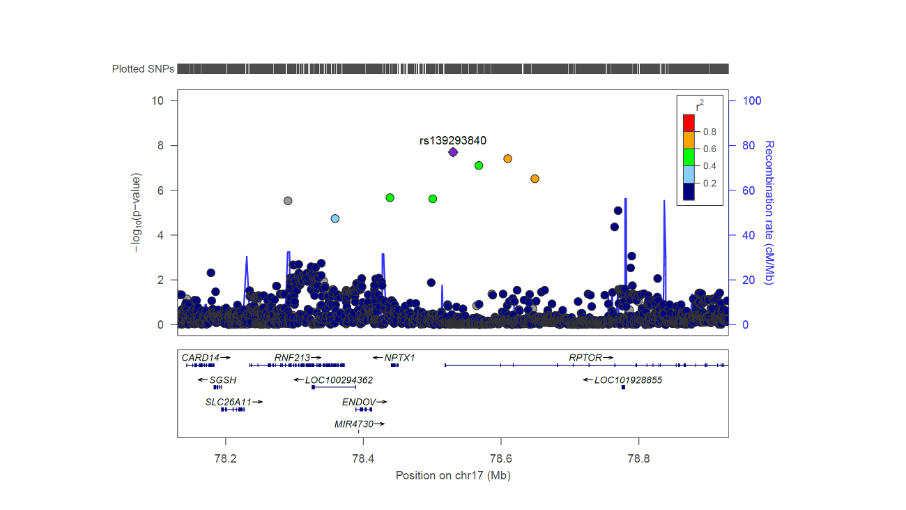


1. DL-CAD


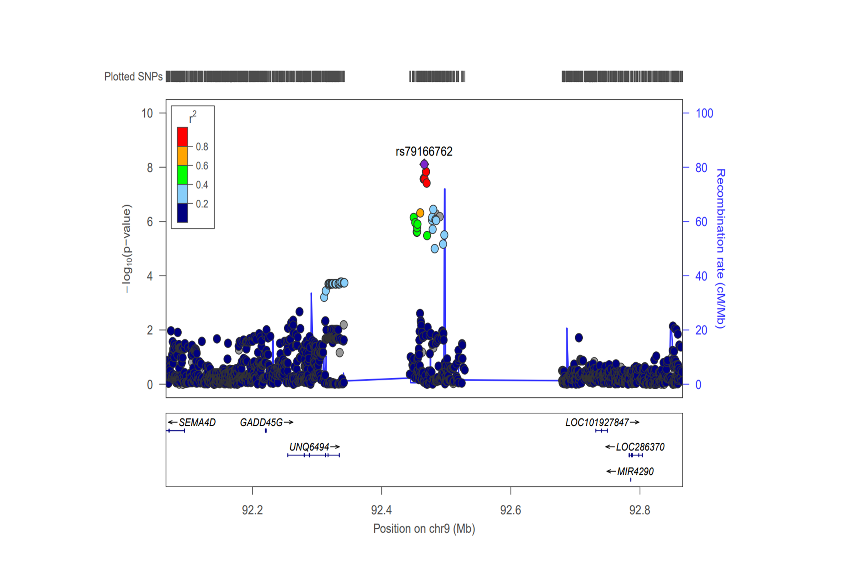


1. DL-CCD


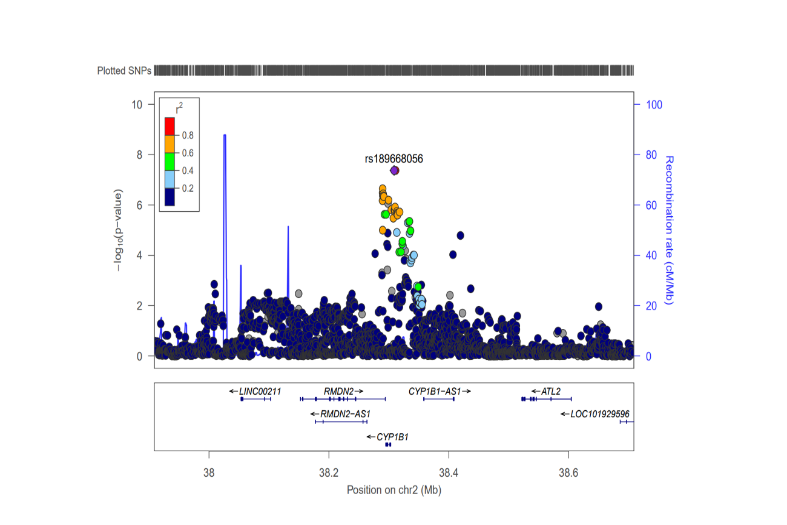


1. DL-IS


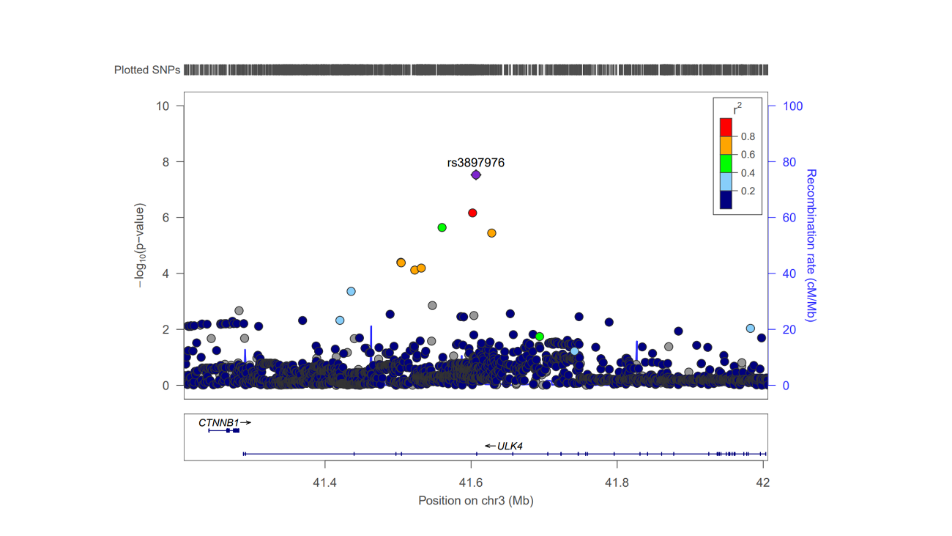

Supplement: Supplementary file 1 — Additional file 1: Table S1. Baseline Characteristics of the Hypertension Study Population. Table S2. Baseline Characteristics of the Diabetes Mellitus Study Population. Table S3. Baseline Characteristics of the Dyslipidemia Study Population. Figure S1. Quantile-quantile plots with genomic inflation values. Figure S2. Regional association plots for lead SNPs, A HTN-CAD B DM-IS C DM-CCD D DL-CAD E DL-CCD F DL-IS. [file 12967_2021_2751_MOESM1_ESM.docx]
